# An Electrochemical Biosensor Analysis of the Interaction of a Two-Vector Phospholipid Composition of Doxorubicin with dsDNA and Breast Cancer Cell Models In Vitro

**DOI:** 10.3390/pharmaceutics16111412

**Published:** 2024-11-02

**Authors:** Lyubov V. Kostryukova, Anastasia S. Serdyukova, Veronica V. Pronina, Victoria V. Shumyantseva, Yulia A. Tereshkina

**Affiliations:** Institute of Biomedical Chemistry, Pogodinskaya Street, 10, Build 8, 119121 Moscow, Russia; kostryukova87@gmail.com (L.V.K.); l.lawliet184@gmail.com (A.S.S.); veronicapunch@mail.ru (V.V.P.); viktoria.shumyantseva@ibmc.msk.ru (V.V.S.)

**Keywords:** doxorubicin, phospholipid nanoparticles, breast cancer, targeted fragments, electrochemical biosensor, DNA

## Abstract

**Objectives:** The main aim of our experiments was to demonstrate the suitability of cell-based biosensors for searching for new anticancer medicinal preparations. **Methods:** The effect of the substance doxorubicin, doxorubicin embedded in phospholipid nanoparticles, and doxorubicin with phospholipid nanoparticles modified by targeting vectors (cRGD and folic acid) on dsDNA and breast cancer cell lines (MCF-7, MDA-MB-231) was studied. **Results:** In the obtained doxorubicin nanoforms, the particle size was less than 60 nm. Our study of the percentage of doxorubicin inclusion showed the almost complete embeddability of the substance into nanoparticles for all samples, with an average of 95.4 ± 4.6%. The calculation of the toxicity index of the studied doxorubicin samples showed that all substances were moderately toxic drugs in terms of adenine and guanine. The biosensor analysis using electrodes modified with carbon nanotubes showed an intercalation interaction between doxorubicin and its derivatives and dsDNA, except for the composition of doxorubicin with folic acid with a linker length of 2000 (NPh-Dox-Fol(2.0)). The results of the electroanalysis were normalized to the total cell protein (mg) and cell concentration. The highest intensity of the electrochemical signals was observed in intact control cells of the MCF-7 and MDA-MB-231 cell lines. **Conclusions:** The proposed electrochemical approach is useful for the analysis of cell line responses to the medicinal preparations.

## 1. Introduction

Breast cancer is the most common type of tumor among women and is the second most common cause of death worldwide [1,2]. The classic treatment for this disease is chemotherapy. Doxorubicin is the best-known and most widely used chemotherapy drug. The mechanism of action of this compound involves the embedding of its anthracycline part in the base pairs of two-stranded DNA and the inhibition of DNA replication and transcription, as well as tumor cell division [3,4]. However, despite its advantages, doxorubicin has a number of side effects; in particular, due to its non-specific distribution in the body, it can cause cumulative dose-dependent cardiotoxicity and can also cause myelosuppression [5,6].

One of the approaches to reduce the side effects of doxorubicin is to use drug delivery systems, including targeted ones. The inclusion of targeting molecules in delivery systems can increase drug accumulation in target tissues and reduce the impact on healthy organs and tissues. Peptides with the RGD (Arg-Gly-Asp) sequence belong to such molecules. They target a cell surface receptor involved in the signaling transduction pathways in the proliferation and metastasis of cancer cells, integrin α_v_β_3_, which is a heterodimeric transmembrane glycoprotein [7,8]. Integrin α_v_β_3_ mediates cell adhesion to the extracellular matrix by recognizing Arg-Gly-Asp (RGD) motifs in various ligands. Integrins play a role in tumor cell migration, which is the main event of the metastatic cascade [9]. In recent years, an increasing number of studies have been conducted using RGD peptides to target integrin α_v_β_3_. The main approach to using a peptide containing an RGD motif is to conjugate it with a linker for subsequent binding to the surface of a nanocarrier with the drug included. The use of nanocarriers with an attached RGD peptide is a promising method for delivering chemotherapeutic agents in the treatment of cancer [10].

In addition to peptides, folic acid is used as a targeting molecule in most studies. The folate receptor (FRα) is overexpressed in breast cancer [11,12,13,14]. A “double-targeting” approach is used to increase the efficiency of drug accumulation in the tumor [15]. This approach involves the use of several ligands on the surface of a single nanocarrier to target different receptors. This approach will help overcome the heterogeneity of antigen expression on different types of tumor cells, increasing the number of potential binding sites and the efficacy of the treatment of cancer.

Various methods are used to analyze chemotherapeutic agents and their effects on targets, such as capillary electrophoresis [16], high-performance liquid chromatography [17], fluorescence spectroscopy [18], and electrochemical methods [19].

Pharmacogenomics is the study of changes in the physicochemical characteristics of DNA and RNA associated with reactions to drugs [20]. Electrochemical analysis of the DNA–drug complex helps evaluate the drug–DNA interaction. DNA biosensors based on the immobilization of DNA probes on a solid surface have been developed for the analysis of antitumor drugs and for studying their interaction with DNA [21]. Electrochemical methods for the analysis of nucleic acids are based on the ability of heterocyclic nitrogenous bases, which are part of nucleotides, to be electrochemically oxidized on the electrode surface. DNA biosensors registering the electrochemical characteristics of the sensors were developed for the analysis of doxorubicin [22,23,24,25,26,27]. To analyze the effect of antitumor drugs in vitro, the characteristics of the drugs themselves before and after the interaction were studied in cell line models [28,29,30].

In our study, a method was developed for the electroanalysis of the breast cancer cell lines MCF-7 and MDA-MB-231 before and after exposure to doxorubicin and its derivatives. The electrochemical profiles of the cell lines allow for the recording of the electroactive components of the cells on the surface of their cell membranes and the evaluation of their changes under the influence of antitumor drugs [31,32]. In this work, we employed this electrochemical approach as a robust method of drug analysis and cell line analysis “in electrode”, which can serve as additional approach for in vitro studies.

The purpose of this study was to evaluate the interaction mechanisms of doxorubicin compositions containing targeting vectors using a previously developed electrochemical method employing DNA bioelectric sensors [15], as well as to study the effect of these components on the breast cancer cell lines MCF-7 and MDA-MB-231.

## 2. Materials and Methods

### 2.1. Characteristics of Doxorubicin Nanocompositions

This study used a doxorubicin nanoform (nanocomposition) (NPh-Dox) and its modifications with targeting conjugates: (a) cRGD peptide (NPh-Dox-cRGD); (b) folic acid, with different linker lengths of 2000 and 3400 (NPh-Dox-Fol(2.0) and NPh-Dox-Fol(3.4)); and (c) two conjugates together (NPh-Dox-cRGD-Fol(2.0) and NPH-Dox-cRGD-Fol(3.4)). The doxorubicin hydrochloride substance (JSC Omutninskaya Scientific Pilot-Industrial Base, Vostochnyi, Russia) was used as a reference. The method of preparation of the resulting phospholipid composition and its modification were described earlier [15].

The drug concentrations are presented in Table 1.

All the obtained doxorubicin nanocompositions (nanoforms) were characterized before the studies by the following physico-chemical parameters: particle size, ζ-potential with a Zetasizer Nano laser correlation spectrometer (Malvern Instruments Ltd., Malvern, UK) with Malvern ZETASIZER 6.20 software, and percentage of inclusion of the doxorubicin substance in phospholipid nanoparticles by ultrafiltration using VivaSpin 500 microfilters (Sartorius AG, Gothengem, Germany) in accordance with the previously described methods [15,33].

### 2.2. Study of the Effect of Doxorubicin Compositions Containing Target Vectors on dsDNA

Electrochemical measurements were performed using an Autolab potentiostat, model AUT128N, with the NOVA2.0 software. We used three-contact electrodes with a graphite working electrode (with an electrode working surface diameter of 2 mm) and an auxiliary electrode, as well as an Ag/AgCl reference electrode.

Three-contact screen-printed graphite electrodes (SPEs) were purchased from ColorElectronics (LLC “ColorElectronics”, Moscow, Russia). This study used the method of non-covalent modification of a screen-printed graphite electrode with carbon nanomaterials. The electrode was modified by applying 2 µL of carbon nanotube (CNT) dispersion in carboxymethylcellulose (Tuball Batt), which was diluted with distilled water in a ratio of 1:5 (SPE/CNT). Double-stranded DNA (dsDNA) isolated from sturgeon sperm was obtained from Sigma-Aldrich (St. Louis, MO, USA), (D3159, Product of USA), M = 10–30 kDa. A DNA concentration of 1.5 mg/mL was used for analysis of DNA and DNA complexes with drugs.

The following reagents were used: monosubstituted potassium phosphate (Reakhim, Dzerzhinsk, Russia), sodium chloride (Reakhim, Dzerzhinsk, Russia), single-walled carbon nanotubes (SWCNTs) at 0.4 wt%, stabilized with carboxymethylcellulose at 0.6 wt% (OCSiAl, https://ocsial.com URL10092024, Differdange, Luxembourg), and double-stranded DNA extracted from sturgeon milk (Sigma-Aldrich, Tokyo, Japan).

The working surface of the SPE was coated with 2 μL of a dispersion of SWCNTs stabilized with carboxymethylcellulose, with a concentration of 0.75 ± 0.05 mg/mL (SPE/CNTs). To prepare this dispersion, 0.02 g of an initial SWCNT dispersion with a concentration of 0.4 wt% was diluted in 100 μL of H_2_O. The electrodes were modified for 30 min at room temperature, followed by pretreatment (four differential pulse voltammetry (DPV) scans within the potential range of 0–1.2 V). Experiments were conducted under aerobic conditions at room temperature in a horizontal mode [24,25].

Samples of 2 µL of the phospholipid compositions were applied to the surface of a carbon nanotube-modified electrode (SPE/CNT). The electrodes were incubated for 30 min at room temperature. Scanning was performed by cyclic voltammetry in the potential range of −1.0 ÷ +1.0 V, with a scanning rate of 0.1 V/s, in an aerobic environment at room temperature. Further analysis was performed by differential pulse voltammetry (DPV) in the potential range of +0.1 ÷ +1.2 V, in an aerobic environment at room temperature. DPV parameters were as follows: modulation amplitude of 25 mV, potential increment of 5 mV, modulation time of 50 ms, and time interval of 0.5 s.

DNA–drug complexes were formed during an incubation for 10 min at room temperature. The DNA–nanoform complex was studied by applying it (at a 1:1 ratio) to the electrode, followed by 5-min incubation. This study was performed using differential pulse voltammetry in the potential range of +0.1 ÷ +1.2 V, in an aerobic environment at room temperature [24,25].

### 2.3. Electrochemical Studies In Vitro

Breast cancer cell lines MCF-7 and MDA-MB-231 were obtained from the American Type Culture Collection (ATCC) and maintained in the IBMC cell culture collection.

The cells MCF-7 and MDA-MB-231 were cultured according to the recommendations specified in the cell culture certificates. The cells were incubated in Dulbecco’s modified Eagle’s medium (DMEM) (PanEco, Moscow, Russia) with 2 mM L-glutamine and 10% FBS (fetal bovine serum) (Capricorne, Ebsdorfergrund, Germany) added, in 25 cm^2^ and 75 cm^2^ culture flasks (Biologyx, Jinan, Shandong, China), at 37 °C, in a humid atmosphere with 5% CO_2_ (CO_2_ incubator, Sanyo, Moriguchi, Osaka, Japan). Versene solution and a 0.25% trypsin solution were used during the passage to detach the cells, adding them to the flask for 2–3 min. The cells were used in this study after 3-18 passages and freezing.

MCF-7 and MDA-MB-231 cells were inoculated (10^6^ cells per well) into 12-well plates (Biologyx, Jinan, Shandong, China) and cultured at 37 °C in an atmosphere with a relative humidity of 95% and 5% CO_2_ for 24 h. The introduced test samples had a concentration of 10 µg/mL (with reference to doxorubicin) and were incubated for 24 h at 37 °C in the CO_2_ incubator. After the incubation, the medium with the introduced samples was removed. The cells were washed three times with Versene solution and detached as described above. The cell suspension was washed twice with phosphate buffered saline (PBS; PanEco, Moscow, Russia) and precipitated in an Elme centrifuge at 1000× *g* for 4 min, followed by removal of the supernatant. The cell precipitate was suspended in 25 µL of potassium phosphate buffer.

The cell suspension (1.6 × 10^5^ cells) was applied to the working electrode (SPE/CNT) and immobilized on it for 40 min, at room temperature in an aerobic environment. After the immobilization, 60 µL of potassium phosphate buffer was applied to the electrode and incubated for 5 min, followed by square wave voltammetry (SWV) in the potential range from 0.0 to +1.0 V, increment 5 mV, amplitude 0.02 V, and frequency 10 Hz.

The obtained results were normalized to protein according to Lowry assay and to the number of cells counted using the ViCell XR analyzer (Beckman Coulter, Brea, CA, USA).

The DNA-mediated electrochemical coefficient of the toxic effect was calculated according to the following formula [34]:

(1) $$T=\frac{I(DNA)}{I_{D}}\times 100\%$$

where *I*(*DNA*) is the oxidation peak current of the heterocyclic base of the control DNA and *I_D_* is the oxidation peak current of the heterocyclic base of the DNA in the complex [DNA–drug].

The calculation was carried out for each heterocyclic base, for each of the drugs used in the work.

### 2.4. Statistical Analysis

Student’s test was used to assess the significance of differences in the electrochemically measured parameters over three measurements. Differences were considered statistically significant at *p* ≤ 0.05. In the figures, the data are presented as the mean value ± standard error relative to the mean value.

## 3. Results

### 3.1. Characterization of the Physico-Chemical Properties of Doxorubicin Nanocompositions

In our earlier work [15], we developed a two-vector phospholipid composition of doxorubicin to increase the accumulation of this therapeutic agent in tumor cells. This composition was an ultrathin emulsion with a particle size of up to 50 nm, and 98% of the doxorubicin was embedded in the nanoparticles. The composition was stable when stored for 48 h. In this series of experiments, which are similar to the previous work [15], the physico-chemical parameters were first evaluated in order to establish the reproducibility of the conditions used to obtain the compositions, which will further assess the reliability of the results obtained.

**Figure 1:**
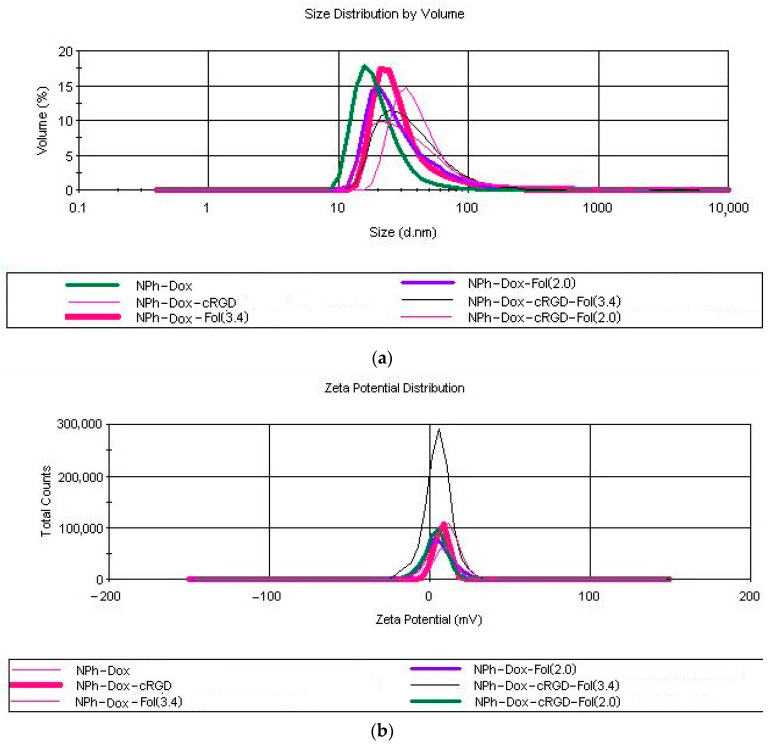
Particle size distribution (**a**) and zeta potential (**b**) in the prepared doxorubicin nanocompositions.

Our study of the particle size of the resulting phospholipid composition and its modifications was carried out using the dynamic light scattering method. In the obtained doxorubicin nanoforms, the particle size was less than 60 nm (~95 ± 5.2% of particles were within this volume distribution) (Figure 1a). The ζ-potential studies were carried out using electrophoretic light scattering. The obtained results of the ζ-potential studies showed that the values of this parameter for all the studied samples were within 10 mV (Figure 1b). Our study of the percentage of doxorubicin inclusion showed the almost complete embeddability of the substance into the nanoparticles for all samples, with an average of 95.4 ± 4.6%.

### 3.2. The Effect of Doxorubicin Compositions Containing Target Vectors on dsDNA

Doxorubicin, as antitumor drug, is widely used in the treatment of a large number of malignancies [30]. However, doxorubicin itself possesses many side effects, such as multidrug resistance and cardiotoxicity [35]. To increase the efficacy of doxorubicin and reduce side effects, extensive research is being conducted to obtain various forms and achieve targeted delivery of the drug. In particular, there is increasing interest in delivery systems with additional functions performed by targeting molecules. Due to their high affinity for cancer cell receptors, peptides containing the sequence RGD, as well as folic acid, are the most commonly used ligands. The mechanism of action of doxorubicin and the manifestation of its toxic action have been studied using various methods. Previously, comparative studies of the interaction and effect of doxorubicin and a composition of doxorubicin with a targeting peptide containing the NGR motif (Asn-Gly-Arg) on dsDNA were carried out using differential pulse voltammetry. A change in the mechanism of interaction in DNA–drug complexes from intercalation (for doxorubicin) to electrostatic (for doxorubicin with NGR) was shown [25].

Previously developed nanoforms of doxorubicin with targeting fragments [15] performed well in vitro in the study of cell accumulation and cytotoxic action.

The effect of the additional targeting fragments of (A) the cRGD peptide (NPh-Dox-cRGD); (B) folic acid, with different linker lengths of 2000 and 3400 (NPh-Dox-Fol(2.0) and NPh-Dox-Fol(3.4)); and (C) two conjugates (NPh-Dox-cRGD-Fol(2.0) and NPh-Dox-cRGD-Fol(3.4)) on the interaction of doxorubicin with DNA was studied by electrochemical analysis using DNA sensors.

**Figure 2:**
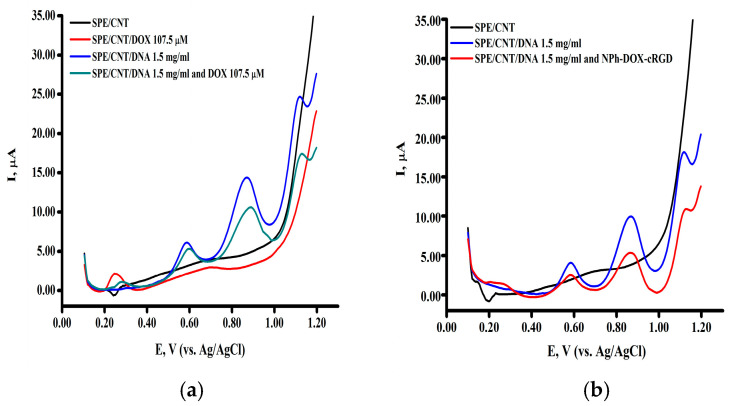
The differential pulse voltammograms (**a**) of the dsDNA complex (1.5 mg/mL) with doxorubicin and (**b**) of the dsDNA complex with the phospholipid composition of doxorubicin NPh-Dox-cRGD. The measurements were performed in the potential range of +0.1 ÷ +1.2 V, in an aerobic environment at room temperature.

Figure 1 shows the differential pulse voltammograms of the complexes of DNA (1.5 mg/mL) and Dox (Figure 2a) and the complexes of DNA (1.5 mg/mL) and NPh-Dox-cRGD (Figure 2b). As follows from the presented voltammograms, a decrease was observed in the intensity of the maximum amplitude of the currents corresponding to the electrochemical oxidation of the DNA bases and a shift in the potentials of the electrochemical oxidation of DNA bases to the positive (anode) region, which indicates an intercalation mechanism of interaction for both doxorubicin and its derivative with the NPH-Dox-cRGD targeting fragment.

**Figure 3:**
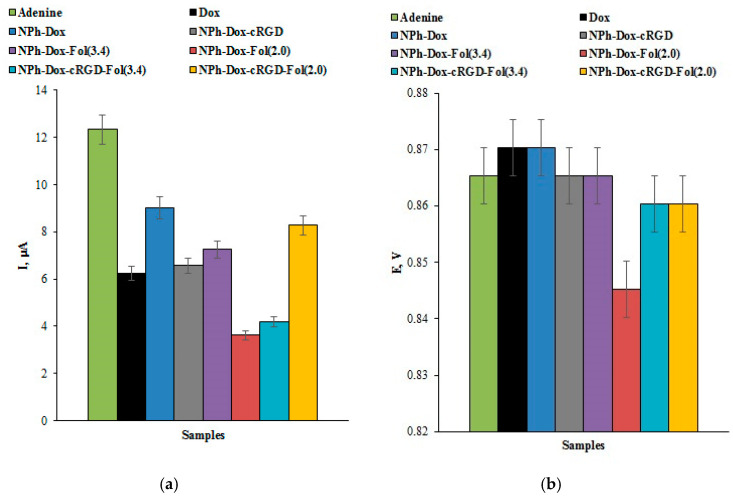
(**a**) The histogram of the effect of doxorubicin and its nanoforms on the oxidation peak current of adenine in dsDNA; (**b**) The histogram of the effect of doxorubicin and its nanoforms on the electro-oxidation potential of adenine. The measurements were performed by DPV in the potential range of +0.1 ÷ +1.2 V, in an aerobic environment at room temperature.

The effect of doxorubicin and the additional targeting fragments (A) of cRGD peptide (NPh-Dox-cRGD); (B) folic acid, with different linker lengths of 2000 and 3400 (NPh-Dox-Fol(2.0) and NPh-Dox-Fol(3.4)); and (C) two conjugates (NPh-Dox-cRGD-Fol(2.0) and NPh-Dox-cRGD-Fol(3.4)) on the intensity of the maximum current amplitude of the DNA bases and the shift in the electrochemical oxidation potentials assessed by differential pulse voltammetry of the doxorubicin nanoform complexes with DNA are shown in Figure 3a,b.

The main conclusion that can be made from the obtained results of the analysis of the complexes of DNA with doxorubicin or its nanoforms with targeting fragments is a decrease in the amplitude of the currents corresponding to the heterocyclic DNA bases as the target for the drugs. The observed difference in the oxidation peak current of dsDNA adenine indicates the interaction of Dox and its derivatives with the dsDNA molecule (Figure 3a) [36]. The decrease in the electro-oxidation intensity of the dsDNA bases corresponds to the formation of an electrochemically less active complex compared with intact dsDNA. The information on the shift in the purine electro-oxidation potential to the negative (cathode) or positive (anode) region helps characterize the DNA drug-binding process. Intercalation binding is associated with a shift in the electrochemical oxidation potentials of the heterocyclic bases to the positive (anode) region, which was shown for doxorubicin (a potential shift of 20 mV) and its nanoforms (Figure 2a,b). The modification of doxorubicin with targeting fragments reduced the intercalation effect (Figure 2b and Figure 3b). This can be explained by the structure of the studied forms containing the substance inside phospholipid nanoparticles and the vectors on the surface of the nanoparticles.

The calculation of the DNA-mediated electrochemical coefficient of the toxic effect [34] showed (Table 2) a decrease in the current for all doxorubicin nanoforms for adenine, thymine, and guanine.

The greatest decrease was observed with the complex with the free doxorubicin substance, as well as the phospholipid composition (NPh-Dox), and the phospholipid composition with the addition of the cRGD peptide (NPh-Dox-cRGD) targeting vectors and folic acid with a linker length of 2000 (NPh-Dox-Fol(2.0)) to the phospholipid nanoform.

The obtained results were used to calculate the DNA-mediated electrochemical coefficient of the toxic effect for the doxorubicin samples (Table 2) to assess the nature of the changes in the toxicity of Dox embedded in phospholipid nanoparticles or following its modification by targeting vectors.

According to the accepted criteria, the drug is considered non-toxic if the toxicity index (T) is greater than 85%; the drug shows a moderate toxic effect if the T is 50 to 85%; and the drug has a pronounced toxic effect if the T is less than 50% [34].

The calculation of the toxicity index of the studied doxorubicin samples showed that all substances were moderately toxic drugs in terms of adenine and guanine, but free doxorubicin, NPh-Dox-Fol(2,0) and NPh-Dox-cRGD-Fol(2.0) were toxic for thymine. The presence of targeting fragments generally decreased the DNA-mediated toxicity of doxorubicin.

### 3.3. Electrochemical Study of the Effect of Doxorubicin Nanoforms on Breast Cancer Cells In Vitro

Breast cancer is the most common type of oncological disease in the female population [1]. Doxorubicin is used as a treatment for this disease. However, its use is limited by serious side effects due to its non-specific distribution in the body. The inclusion of a chemotherapeutic agent in delivery systems, including targeted ones, decreases its negative side effects [8,14].

The MTT assay is conventionally used to determine the cytotoxic effect of drugs on cells [37]. However, the study of the cytotoxic effect of new nanoparticle-based drugs using the MTT assay can be somewhat difficult, mainly due to the uncontrolled contribution of nanoparticles to the total absorbance of the solution in the plate well. This effect may be related to the aggregation of nanoparticles in the incubated culture medium. In addition, nanoparticles can bind to the molecules of a dye or other reagent used to determine the viability of cells, thus affecting the results of the measurement. Thus, the use of several methods will avoid possible errors and allow for a more precise determination of the effect of developed nanoparticle-based drugs on tumor cells.

The use of electrochemical methods for the analysis of medicinal products helps select the optimal conditions for drug studies and their impact on cell models to ensure high sensitivity and selectivity [19].

The electrochemical “molecular fingerprints” of cell lines allow for the recording of the electroactive components of cells on the surface of cell membranes and the evaluation of their impairment or change when exposed to antitumor drugs.

The MDA-MB-231 cell line, according to the literature data [38,39], shows both integrin α_v_β_3_ and folate receptor (FRα+) expression; the MCF-7 line shows no expression of integrin α_v_β_3_ [40], while expressing the folate receptor [41].

**Figure 4:**
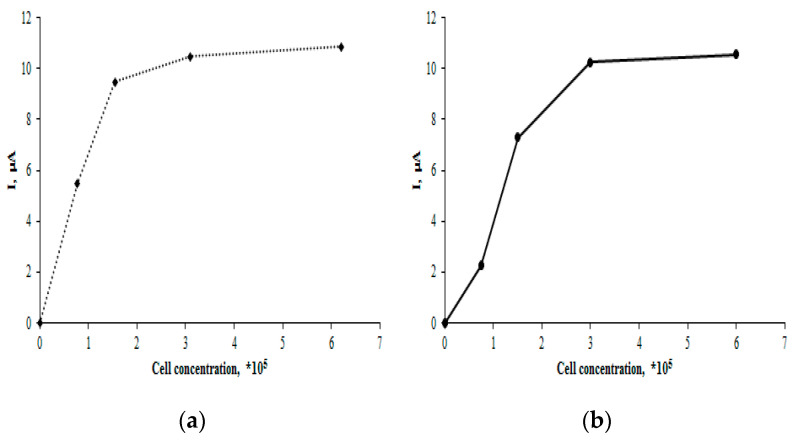
Current signal intensity vs. tumor cell concentration: (**a**) MCF-7 and (**b**) MDA-MB-231.

The electrochemical analysis of the breast cancer cell lines MCF-7 and MDA-MB-231 was performed using square wave voltammetry in the potential range of 0.0 to +1.0. Square wave voltammetry (SWV) was chosen as the most sensitive method, which registered the electrochemical signatures of the MCF-7 and MDA-MB-231 cell lines. The cells were immobilized on SPE/CNT electrodes. The results demonstrated that the oxidation peak current of MCF-7 cells was dependent on the logarithm of the cell concentration, which is consistent with the literature data [42,43,44]. The presented data (Figure 4a) show that the electro-oxidation peak current increased with the cell concentration, reaching a plateau at the cell concentration of 6 × 10^5^. A similar relationship was observed for the MDA-MB-231 cell line (Figure 4b).

**Figure 5:**
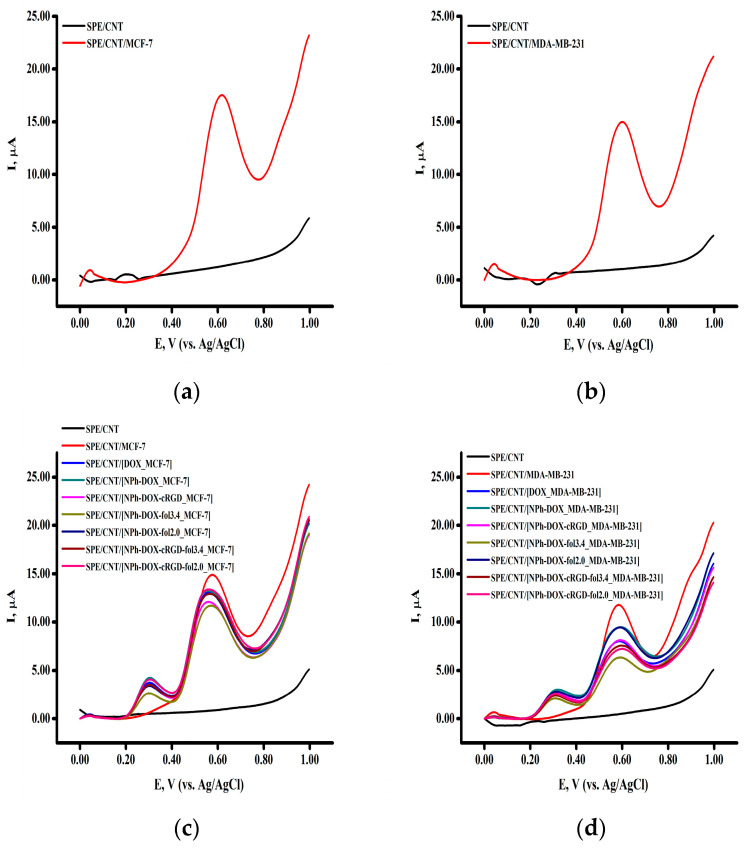
Square wave voltammogram of cells: (**a**) MCF-7; (**b**) MDA-MB-231; cells treated with doxorubicin and its nanoforms (**c**) MCF-7; and (**d**) MDA-MB-231. Concentration of doxorubicin and its nanoforms is 10 µg/mL. Study was performed by square wave voltammetry in the potential range of 0 to + 1.0 V. Peak intensities were normalized per cell/electrode.

In further studies of the effect of doxorubicin and its nanoforms on breast cancer cell lines, 5 × 10^4^ cells were applied to the electrode. Figure 5a,b shows the voltammograms of the MCF-7 and MDA-MB-231 cells obtained by SWV.

To study the effect of the compositions with targeting fragments on MCF-7 and MDA-MB-231 breast cancer cell models, the cells were incubated with samples for 24 h. The doxorubicin concentration in all experiments was 10 µg/mL. Upon incubation of the MCF-7 cells (Figure 5a) with doxorubicin or its phospholipid nanoforms, the highest signal intensity was observed in the control cells (Figure 5b). The interaction of the cells with doxorubicin or its nanoforms having targeting fragments led to a decrease in the intensity of the currents corresponding to the electrochemical profile of the cell lines.

**Figure 6:**
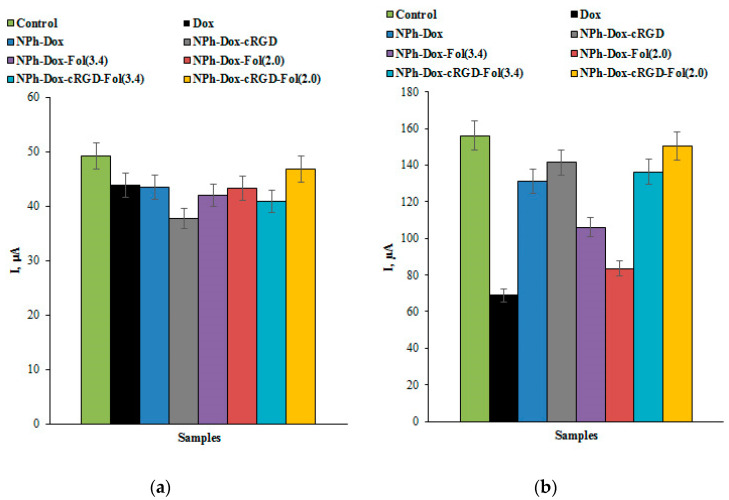
The histograms comparing the intensity of the oxidation peak currents of the cells: (**a**) MCF-7 and (**b**) MDA-MB-231 during incubation with doxorubicin and its phospholipid compositions. The currents were normalized for the concentration of total protein in the cells.

To quantify the effect of the drugs on the cell lines, the cell protein content was determined by the Lowry assay. The current intensity in the electrochemical analysis of the MCF-7 and MDA-MB-231 cell lines was associated with the normalized data obtained for protein content of the sample (Figure 6a,b).

The results of studies of the MCF-7 cell line (Figure 6a) showed the highest signal intensity for the control sample and the lowest signal intensity for the cells incubated with doxorubicin in phospholipid nanoparticles with the cRGD peptide (NPh-Dox-cRGD).

No expression of integrin α_v_β_3_ is observed in the MCF-7 line [39], while the folate receptor is expressed. However, the greatest decrease in the intensity of the signals of the electrochemical profile of the MCF-7 cells was observed with NPh-Dox-cRGD, which has a fragment targeted specifically to integrin α_v_β_3_. This may be due to the rate of release of doxorubicin from the phospholipid nanoparticle.

The MDA-MB-231 cell line, according to the literature data [38,39], shows expression of both integrin α_v_β_3_ and the folate receptor (FRα+). With the MDA-MB-231 cell line (Figure 6b), the lowest signal intensity in terms of mg of protein in the sample was observed in the cells treated with doxorubicin, which do not have targeting fragments.

However, we obtained more pronounced effects of Dox derivatives on cell lines of breast cancer using cell concentration as a reference method (Figure 5a,b). In all experiments with cell lines, we used approximately the same concentration of cells (which was measured on a ViCell XR analyzer), such as 4.4 ± 0.3 MCF-7 cells/electrode and 1.9 ± 0.2 MDA-MB-231 cells/electrode. Using this more correct approach, we can see from Figure 5a,b that the most intense decline in the peak current corresponded to the MCF-7 cells, which express the folate receptor, and this response was registered for the Dox derivative NPh-Dox-Fol(3.4). In the case of the MDA-MB-231 cell line, which possesses both integrin α_v_β_3_ and folate receptor (FRα+) expression, the most intense decline in the peak current corresponding to the cell response was registered for the Dox derivatives NPh-Dox-cRGD-Fol(3.4), NPh-Dox-Fol(3.4), and NPh-Dox-cRGD-Fol(2.0). In these experiments, we register the clear influence of vector systems on the activity of Dox derivatives on the cell lines.

We can conclude that for the quantitative calculation of drug effects on cells, electrochemical parameters are more correct to normalize to the per cell concentration used in experiments.

The electrochemical studies showed that this method could be used in preclinical and clinical studies when analyzing the pharmacological activity of drugs on cell models of diseases. Electrochemical methods help evaluate and compare the effect of various nanoforms of a drug on cell cultures, conduct primary screening of the activity of the studied compounds, and work out the optimal interaction conditions in the cell/potential drug system.

## 4. Discussion

The use of delivery systems helps decrease the side effects of drugs, thereby increasing their efficacy. Targeting molecules deliver the drug directly to the tumor, reducing toxic effects on surrounding healthy tissues/organs. Targeted cyclic peptides containing the RGD motif have affinity for integrin α_v_β_3_, which plays a crucial role in tumor invasion, migration, and metastasis [8,10]. The cyclic peptide cRGD is a promising agent for delivering chemotherapy drugs to the tumor. Small molecules, in particular folic acid targeting the folate receptor, which is overexpressed on the surface of tumor cells, can also be used as a targeting agent [11]. The use of multiple targeting agents can increase the effectiveness of doxorubicin delivery to the tumor as multiple receptors are targeted. The obtained composition of doxorubicin embedded in phospholipid nanoparticles with the cRGD peptide and folic acid (NPh-Dox-cRGD-Fol(3.4)) demonstrated a good potential in breast cancer model studies in vitro [15]. Electrochemical analytical methods allow for an alternative analysis of medicinal products. The mechanism of action and toxicity of doxorubicin can be evaluated using the developed electrochemical method on dsDNA by differential pulse voltammetry. The shift in the electro-oxidation potential to the negative (cathode) or positive (anode) region helps characterize the DNA drug-binding process [45,46]. The electrochemical studies of doxorubicin nanoforms showed a shift in the electrochemical oxidation potentials of the nucleobases to the positive (anode) region, while the equipment of nanoparticles with targeting fragments reduces the intercalation effect. The calculated toxicity indices (for adenine and thymine) revealed that the phospholipid nanoforms of doxorubicin are moderately toxic [24,25]. The thymine toxicity index showed that the free substance, the phospholipid nanoform with folic acid with a linker length of 2000 (NPh-Dox-Fol(2.0)), and the two-vector composition of doxorubicin (NPh-Dox-cRGD-Fol(2.0)) have a toxic effect. Thus, the data obtained indicate a decrease in the DNA-mediated toxicity of doxorubicin in nanoparticles with the use of targeting fragments.

In addition, this method can be used to evaluate the effects of a drug on a cell model, which has been demonstrated by several authors [28,29,30]. For the MCF-7 breast cancer cell line, the cell oxidation peak current was shown to be dependent on the logarithm of the cell concentration [42,44]. A study on the MCF-7 cell line showed the greatest decrease in the intensity of the electrochemical profile signals for the nanoform with the cRGD peptide (based on the protein concentration as determined by the Lowry assay). With the MDA-MB-231 cell line, the lowest signal intensity was observed for free doxorubicin and its nanoform without targeting fragments. However, under the normalized data per cell concentration, the most intense decline in the peak current of the MCF-7 cell response was registered for NPh-Dox-Fol(3.4), and in the case of MDA-MB-231 cells, it was registered for NPh-Dox-cRGD-Fol(3.4), NPh-Dox-Fol(3.4), and NPh-Dox-cRGD-Fol(2.0). Based on our preliminary experimental data, we assume that electrochemical cell-based biosensors are effective instruments for searching for and analyzing new drug derivatives. In further studies, for the quantitative calculation of drug effects on cells, electrochemical parameters must be normalized per the cell concentration used in the experiments.

## 5. Conclusions

The dsDNA–electrochemical biosensors have great advantages to investigate the mechanisms of drug–dsDNA interactions, due to the sensitivity of the method, selectivity of the analysis, and detection of the changes of the dsDNA structure, reflecting the influence of substances on the electrooxidation ability of the nucleobases. Doxorubicin and doxorubicin embedded in phospholipid nanoparticles demonstrated an intercalative mechanism of interaction, which was confirmed by the shifts in the oxidation potentials of the heterocyclic bases of the DNA molecule. Doxorubicin, doxorubicin with phospholipid nanoparticles modified by targeting vectors cRGD, and doxorubicin embedded in phospholipid nanoparticles with folic acid with a variable linker length of 2000 (NPh-Dox-Fol(2.0)) resulted in slight effects on the shifts in the DNA oxidation potentials. This phenomenon may be dealing with the charged targeting fragments of drugs. However, all studied doxorubicin compositions clearly have shown a diminution in the intensity of the maximum current amplitude of the electrochemical oxidation of DNA bases. The DNA biosensor is an additional and efficient analytic tool, which provides information on the influence of a drug or its pharmaceutical compositions on DNA.

An electroanalytical approach was used for the investigation of the effect of doxorubicin and its compositions on breast cancer cell lines (MCF-7 and MDA-MB-231). For the adequate calculation of the effect of the drugs on these cell lines, we normalized the electrochemical parameters to the total cell protein (mg) and cell concentration. All studied compositions reduced the peak intensities corresponding to the electrochemical profiles of the MCF-7 and MDA-MB-231 cell lines as models of breast cancer. Each derivative of doxorubicin needs the selection of conditions and an individual protocol of experiments for its interaction with cells and for the most effective anticancer action. Based on the results obtained, we can conclude that an electrochemical cell-based biosensor is a promising method for searching for new medications and investigating their influence on model cell systems.

## Figures and Tables

**Table 1:** Characteristics of phospholipid nanoforms of doxorubicin.

№	Samples	Concentration by Dox, µg/mL
1	Doxorubicin hydrochloride substance (Dox)	124.7
2	NPh-Dox	119.0
3	NPh-Dox-cRGD	122.6
4	NPh-Dox-Fol(3.4)	126.5
5	NPh-Dox-Fol(2.0)	113.7
6	NPh-Dox-cRGD-Fol(3.4)	123.1
7	NPh-Dox-cRGD-Fol(2.0)	118.0

**Table 2:** The DNA-mediated electrochemical coefficient of the toxic effect of the phospholipid compositions of doxorubicin.

Samples	Toxicity Coefficient (T), % *		
	A	T	G
Dox	65.7	44.8	63.7
NPh-Dox	72.9	67.7	65.5
NPh-Dox-cRGD	65.7	55.1	68.1
NPh-Dox-Fol(3.4)	65.7	44.1	70.9
NPh-Dox-Fol(2.0)	62.4	50.7	52.7
NPh-Dox-cRGD-Fol(3.4)	73.8	74.1	65.1
NPh-Dox-cRGD-Fol(2.0)	62.5	38.1	64.9

* Average values from three experiments are given.

## Data Availability

The data are available on reasonable request from the corresponding author.
